# A New Electrophysiological Approach to Sleep Microstructure: The Gülhane Classification of K-Complex Subtypes in Narcolepsy Type 1 and Type 2

**DOI:** 10.3390/life16091556

**Published:** 2026-09-16

**Authors:** Volkan Tekin, Mehmet Koçer, Esra Ünverdi-Bıçakçı, Fulya Şahin, Bülent Devrim Akçay, Sinan Yetkin

**Affiliations:** 1 Department of Physiology, Gülhane Faculty of Medicine, University of Health Sciences, 06010 Ankara, Türkiye; 2 Sleep Research Center, Department of Psychiatry, Gülhane Education and Research Hospital, 06010 Ankara, Türkiye; kyocery@gmail.com (M.K.); esraunverdi@gmail.com (E.Ü.-B.); fullyasahin@gmail.com (F.Ş.); bulentdevrim.akcay@sbu.edu.tr (B.D.A.); sinan.yetkin@sbu.edu.tr (S.Y.)

**Keywords:** narcolepsy, K-complex, sleep microstructure, polysomnography, K-complex morphology

## Abstract

**Study Objectives:** While the pathophysiology of Narcolepsy Type 1 (NT1) involves hypocretin deficiency, the microstructural electrophysiological features that distinguish NT1 from Narcolepsy Type 2 (NT2) remain incompletely defined. This study evaluated sleep microstructure with a six-subtype K-complex (KC) categorization (Gülhane Classification). **Methods:** Retrospective polysomnography from the December 2016–January 2026 clinical archive was reviewed. K-complex scoring defined the analyzed sample: NT1 *n* = 18, NT2 *n* = 13, and simple-snoring controls with AHI < 5 (*n* = 15). Complete overnight PSG summaries were available for NT1 *n* = 16 and NT2 *n* = 13. Six visually defined KC subtypes (Isolated-KC, KC-Burst, KC-Sigma, KC-Delta, Polyphasic-KC, and Polyphasic-KC-Sigma) were compared across Descendant, Intermediate, and Ascendant portions of the NREM cycle. **Results:** Compared with controls, NT1 showed lower Isolated-KC and KC-Sigma counts across cycle periods (*p* < 0.05). NT1 also had higher wake after sleep onset (WASO; 34.7 ± 21.1 min) and lower sleep efficiency (90.7 ± 5.4%) than NT2. NT1–NT2 differences that reached *p* < 0.05 were KC-Delta in the Intermediate (*p* = 0.015) and Descendant (*p* = 0.030) periods, Isolated-KC in the Intermediate period (*p* = 0.039), and KC-Sigma in the Descendant period (*p* = 0.041). **Conclusions:** In this retrospective cohort, morphological KC subtyping showed a coherent reduction in Isolated-KC and KC-Sigma in NT1. The findings are descriptive and require prospective validation; they do not constitute a standalone diagnostic test.

## 1. Introduction

The K-complex (KC) is a distinctive graphoelement of non-rapid eye movement (NREM) stage 2 (N2) sleep, first described in the 1930s by Loomis and colleagues [1]. In sleep electroencephalography (EEG) it is a high-voltage, biphasic slow wave. It is not a sharp wave: by conventional EEG terminology a sharp wave lasts <200 ms, whereas a slow wave lasts >200 ms, and the KC belongs to the latter class [2,3]. Operationally, we identified a KC as a well-delineated surface-negative slow wave of at least 75 µV, lasting at least 0.5 s, followed by a surface-positive component. In standard sleep-EEG polarity, the initial deflection is upward (negative). These duration landmarks were applied visually for event identification, not as stored measurements: from the onset of the negative phase to the return of the trailing positive phase to baseline, in at least two concurrent EEG derivations [2]. KCs arise predominantly over frontocentral regions [3,4,5].

KCs may be evoked by an identifiable external stimulus or occur without an obvious trigger (spontaneous KC). Some authors have proposed that apparently spontaneous KCs are still reactions to unnoticed internal events such as snoring, body movement, or a change in respiration [6]. Two functional readings coexist: the KC as a brief micro-arousal, and the KC as a sleep-protective “sentinel” that limits cortical responsiveness to perturbation. Halász summarized this duality as a “Janus face” [7]. Cortically, the waveform corresponds to a widespread down-state after a brief excitatory up-state [7,8].

The KC is a defining graphoelement of N2 in the Rechtschaffen and Kales system [3]. That historical staging role is related to, but not the same as, its place in the cyclic alternating pattern (CAP): KC-related activity contributes to CAP phase A1, the CAP component with the least autonomic activation [9,10]. Qualitatively, KCs may appear as a single waveform, as a KC coupled to a sleep spindle, or as successive “massive” KCs (runs of two or more high-voltage KCs). Massive KCs are distinguished from stage N3 on epoch grounds: they remain scored as N2 when 0.5–2 Hz activity occupies <20% of the 30 s epoch, whereas N3 requires ≥20% of the epoch to consist of 0.5–2 Hz waves ≥75 µV (AASM version 3.0).

Previous KC classifications differ in purpose and in what they cannot resolve. Johnson and Karpan (1968) grouped spontaneous KCs into three shape-and-accompaniment classes (A–C) but did not apply mutually exclusive overnight labels or cycle-phase assignment [11]. Paiva and Rosa (1991) described six morphological variants of isolated KCs; accompanying spindle or delta activity was not used as a primary classifier [12]. Ujszászi and Halász (1986) defined nine evoked-KC subtypes from post-stimulus peak latencies (for example N300–P400, N300–P800, N600–P800); this approach requires controlled stimulation and is not readily applied to spontaneous all-night recordings [13]. The CAP framework defines K-alpha and K-sigma within A1 and addresses cyclic arousal organization rather than one-to-one morphological assignment of each KC [10]. The Gülhane Classification used here is also morphological. It is not presented as universally superior to those systems. It is an operational visual scheme for spontaneous overnight KCs, with mutually exclusive labels (priority hierarchy) and cycle-phase resolution, intended for routine clinical polysomnography.

1. Johnson and Karpan (1968): three shape-and-accompaniment groups (A–C); no mutually exclusive overnight or cycle-phase rule [11].
2. Paiva and Rosa (1991): six morphological variants of isolated KC; accompanying rhythms were not the primary classifier [12].
3. Ujszászi and Halász (1986): nine evoked-KC latency/amplitude subtypes; requires controlled stimulation [13].
4. CAP: K-alpha and K-sigma described within phase A1; addresses cyclic arousal organization rather than one-to-one KC labeling [10].

Reductions in KC density—that is, in the number of KCs rather than in anatomical “brain density”—have been reported in Alzheimer disease, schizophrenia, and aging [14,15,16,17,18]. Epileptiform discharges may be preceded by KC-like waveforms [4,7]. In insomnia, a uniform KC deficit has not been established; counts may vary with sleep fragmentation [19,20]. In obstructive sleep apnea and upper-airway resistance, KC morphology itself is altered (shorter duration and lower amplitude); this refers to the EEG waveform, not to the vocal folds [4]. In the same patients, alpha-arousal activity may replace delta-coupled forms [21]. Aging alone also lowers KC density and amplitude [4].

Narcolepsy is a chronic neurological disorder with excessive daytime sleepiness, sleep paralysis, hypnagogic or hypnopompic hallucinations, disrupted nocturnal sleep, and sleep-onset REM periods (SOREMPs) [22,23]. Narcolepsy type 1 (NT1) is defined by cataplexy and/or cerebrospinal-fluid (CSF) hypocretin-1 deficiency; narcolepsy type 2 (NT2) lacks cataplexy and usually has preserved hypocretin-1 [23,24]. NREM microstructure is fragmented in narcolepsy, with reduced CAP A1 activity and altered delta-band and spindle dynamics [25,26,27]. Direct morphological KC subtyping across NT1 and NT2 remains limited. The objective of this study was to compare the six Gülhane KC subtypes among NT1, NT2, and controls, and across Descendant, Intermediate, and Ascendant portions of the NREM cycle.

Prior EEG work has reported reduced CAP A1 activity, lower N2 delta power, and subtype-dependent spindle patterns in narcolepsy [25,26,27,28].

## 2. Material and Method

This retrospective study was approved by the Gülhane Scientific Research Ethics Committee (decision 2026/240). Polysomnography recordings of patients evaluated between December 2016 and January 2026 in the Sleep Research Center archive of Gülhane Education and Research Hospital were reviewed. Recordings were obtained on Grass 4.5, Compumedics, or Philips systems and were staged by at least two scorers on the native review software according to the American Academy of Sleep Medicine (AASM) version 3.0 manual. NT1 and NT2 diagnoses followed ICSD-3 clinical criteria (narcolepsy with cataplexy versus narcolepsy without cataplexy). CSF hypocretin-1 was not obtained systematically in this archive and was therefore not used as an inclusion criterion; this is stated as a limitation. Files were then screened against the inclusion and exclusion criteria below.

The study comprises three groups:

1. Narcolepsy Type 1;
2. Narcolepsy Type 2;
3. Control.

A G*Power 3.1 estimate for a two-sided nonparametric group comparison (α = 0.05; power = 0.80) indicated 21 participants per group; the achieved K-complex-scored sample was smaller. Group *n* throughout is the K-complex-scored set: NT1 *n* = 18, NT2 *n* = 13, and control *n* = 15. Overnight architecture comparisons used the subset with complete PSG summaries: NT1 *n* = 16 and NT2 *n* = 13. Period-wise *n* is smaller when a night lacked a scorable Descendant, Intermediate, or Ascendant segment (Descendant 17/12/15; Intermediate and Ascendant 15/13/15). Gender matching was attempted, but the narcolepsy recordings were entirely male; the 15 controls included 12 men and 3 women. Body-mass index was not systematically recorded in the archive.

Criteria for inclusion:

1. Narcolepsy recordings: adolescents and adults aged 14–65 years with an ICSD-3 diagnosis of NT1 or NT2 and AHI < 5 (the planned adult window was 18–65 years; one NT1 participant in the scored sample was younger than 18 and is discussed as a limitation).
2. Control recordings were not required to have narcolepsy. They were patients referred for suspected apnea who had simple snoring, AHI < 5, and no ICSD-3 narcolepsy diagnosis. One control was younger than 18 years. Residual snoring was accepted as a possible KC trigger and is discussed as a limitation.

Criteria for exclusion:

1. Epilepsy;
2. Using CPAP;
3. Individuals with a history of cerebrovascular diseases (stroke, etc.);
4. Individuals with multiple sclerosis or neurodegenerative diseases;
5. Patients with parasomnia;
6. Those undergoing treatment for psychotic disorders;
7. Individuals receiving treatment for anxiety or depression;
8. Use of REM-suppressing medications.

## 3. K-Complex Analysis

Sleep stages were scored first according to AASM version 3.0. KCs were then identified visually in N2 epochs only. The laboratory used the AASM-recommended EEG montage referenced to the contralateral mastoid (F3-M2, F4-M1, C3-M2, C4-M1, O1-M2, and O2-M1), together with left and right electro-oculography (EOG) and chin electromyography. Exported figure panels do not print channel names; captions state this laboratory montage. A 1 s calibration bar was added to the representative exported traces from the software 1 s grid; a voltage calibration pulse was not present in the exports. Analyses used the clinically displayed, hardware-filtered traces (typical EEG display bandwidth 0.3–35 Hz; sampling rate at least 200 Hz). Unfiltered raw amplifier data were not re-exported. Epochs or segments with movement, electrode, sweat, or respiratory artifacts that obscured the KC waveform were rejected before scoring. Sigma (sleep-spindle) activity was recognized visually as an 11–16 Hz burst of at least 0.5 s; delta activity accompanying a KC was recognized visually as 0.5–4 Hz waves. No spectral power analysis was used for subtype assignment.

After review of the systems above, KCs were assigned to one of six Gülhane subtypes:

1. Isolated-KC;
2. KC-Burst;
3. KC-Sigma;
4. KC-Delta;
5. Polyphasic-KC;
6. Polyphasic-KC-Sigma.

All scored events were slow waves, not sharp waves: a negative (upward) slow component of at least 75 µV and at least 0.5 s, followed by a positive (downward) component, visible in at least two EEG derivations. For identification, the negative phase was taken from the first clear deviation from pre-event baseline to the return through baseline, and the positive phase from that baseline crossing to the return of the after-going wave to baseline; total duration is the sum of the two phases. The 0.5 s rule applies to the negative slow phase, not to the positive phase alone. These landmarks were applied visually as inclusion criteria; per-event phase durations were not stored or analyzed as endpoints. Because a single KC can morphologically satisfy more than one accompanying-wave rule, each event was assigned to only one subtype by the following priority: Polyphasic-KC-Sigma > Polyphasic-KC > KC-Sigma > KC-Delta > KC-Burst > Isolated-KC.

### 3.1. Isolated-KC

Isolated-KC was defined as a single KC that was not accompanied, in the 2 s before or after the event, by a sleep spindle (11–16 Hz), a delta wave (0.5–4 Hz), another KC, or alpha/beta activity. The mention of delta, spindle, and alpha/beta activity in this rule is exclusionary: those features assign the event to another subtype or exclude it from Isolated-KC.

### 3.2. KC-Burst

KC-Burst was defined as at least two KCs occurring within 3 s, without intervening alpha activity. Alpha-containing KC sequences were not counted in this subtype. We did not quantify the number of KCs that carried alpha as a separate category.

### 3.3. KC-Sigma

KC-Sigma was defined as a KC accompanied by a sleep spindle (not a “sleep needle”): an 11–16 Hz burst of at least 0.5 s, occurring within 2 s before the KC, overlapping the KC, or within 2 s after the KC. If a spindle and a qualifying delta sequence were both present, the priority rule assigned the event to KC-Sigma rather than KC-Delta.

### 3.4. KC-Delta

KC-Delta was defined as a KC followed or overlapped by a delta sequence longer than 2 s, containing at least two 0.5–4 Hz waves whose amplitude was at least one-third of the background activity in at least two derivations. A single trailing slow wave that did not meet this duration and count rule was not sufficient for KC-Delta.

### 3.5. Polyphasic-KC

Polyphasic-KC (previously labeled KC-Vertex or KC-VSW) was defined as a KC with one or more additional high-voltage deflections immediately before or on the initial negative phase, producing a triphasic or polyphasic contour. These extra deflections are treated as part of a KC variant, as already illustrated in the Rechtschaffen and Kales atlas, rather than as proof that a vertex sharp wave and a KC were generated together. We do not claim that the preceding wave is a true vertex sharp wave; the earlier “vertex” label is therefore withdrawn.

### 3.6. Polyphasic-KC-Sigma

Polyphasic-KC-Sigma was defined as a polyphasic KC that also met the KC-Sigma spindle rule. Its physiological meaning remains uncertain. It was not the least frequent subtype: KC-Delta had the lowest totals in NT1 and overall.

Scored KCs were then grouped by sleep-cycle period as well as by diagnosis. Descendant (D) is the NREM portion of a cycle in which sleep deepens toward N3. Ascendant (A) is the NREM portion between the deepest N3 of that cycle and the following REM period. Intermediate (I) comprises remaining N2 segments when N3 is fragmented, excluding A. These labels were applied only to NREM (mainly N2) segments. They do not require a normal N1–N2–N3 sequence at sleep onset. In narcolepsy, sleep-onset REM is common: a cycle that began with REM was not given a D period until the first subsequent NREM segment that deepened toward N3; if no N3 occurred, remaining N2 was labeled I by consensus of the two scorers. Nights without any scorable N2 KC were not entered into subtype counts. Not every recording contained all three periods: some nights lacked a scorable D segment (no subsequent deepening toward N3 after sleep-onset REM), and some lacked I or A because N3 was absent or N2 was too fragmented to assign that label. The global K-complex *n* is therefore the number of participants with at least one scored N2 KC, whereas each period-wise *n* is the subset of those participants who had that period on the analyzed night. This is why period *n* is smaller than the group *n* (NT1 18 globally vs. 17/15/15 in D/I/A; NT2 13 vs. 12/13/13; controls 15 in all three periods).

Statistical analyses were conducted in Python 3.9.6 (Python Software Foundation, Wilmington, DE, USA) with pandas 2.3.3, NumPy 2.0.2, SciPy 1.13.1, matplotlib 3.9.4, and seaborn 0.13.2. Continuous variables are expressed as mean ± standard deviation or median with interquartile range (IQR), whereas categorical variables are presented as counts and percentages. Non-parametric tests were employed for count data and data that fail to satisfy parametric assumptions. To compare groups, the Kruskal–Wallis test was utilized, with the Mann–Whitney U test applied specifically for PSG comparisons between NT1 and NT2. No imputation procedures were implemented for missing data; all analyses were based on available observations. Statistical significance was defined as a two-sided p-value less than 0.05. Pairwise period tests used available observations only: a participant contributed to a D, I, or A contrast solely when that period was present and scorable. Consequently the period-wise *n* (Descendant NT1/NT2/control = 17/12/15; Intermediate and Ascendant 15/13/15) is not a second cohort and is not missing data in the usual sense; it is the same KC-scored group with unequal representation of D, I, and A across nights. Pairwise p-values are uncorrected two-sided tests and were not adjusted for the number of subtype-by-period comparisons.

## 4. Results

### 4.1. Participants and Clinical Characteristics

The K-complex-scored sample comprised NT1 *n* = 18, NT2 *n* = 13, and control *n* = 15. NT1 participants were all male, with mean age 25.4 ± 7.0 years in those with a recorded age (*n* = 17; range 15–41); one was younger than 18 years. Where duration of illness was recorded (*n* = 14), mean duration was 8.6 ± 4.8 years (range 3–16). NT2 participants were all male, with mean age 31.0 ± 7.6 years (range 22–46) and mean duration 9.8 ± 6.6 years (*n* = 13; range 1.5–20). Controls (*n* = 15) included 12 men and 3 women; mean age was 25.6 ± 7.1 years in those with a recorded age (*n* = 11; range 17–39). All analyzed recordings had AHI < 5. Controls were evaluated for suspected apnea and had simple snoring without OSA. Severe sleep-disordered breathing was therefore excluded, but residual snoring—which can itself provoke KCs—cannot be ruled out, particularly in the control group. Current use of REM-suppressing medication, antipsychotic medication, or antidepressant medication was an exclusion criterion; a complete historical treatment inventory (stimulants, sodium oxybate, pitolisant, or prior antidepressants) was not uniformly available in the archive. BMI was not systematically recorded. CSF hypocretin-1 was not measured for this study.

### 4.2. Global KC Subtype Comparisons

**Figure 1:**
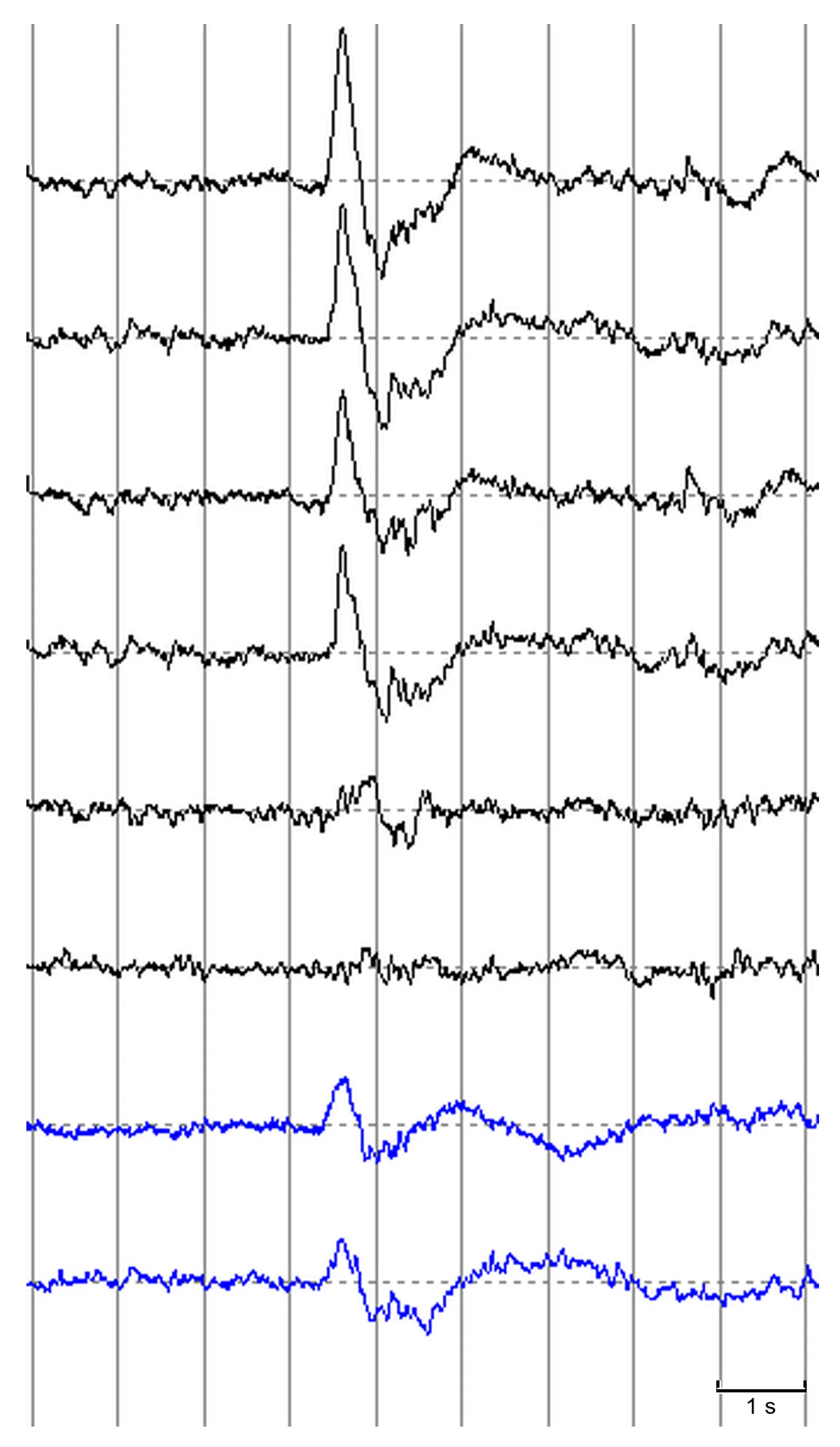
Isolated-KC. Representative N2 excerpt. Laboratory montage (AASM-recommended; channel names are not printed on the export): F3-M2, F4-M1, C3-M2, C4-M1, O1-M2, and O2-M1 (black traces), with left and right EOG (blue traces). The scored event is a single high-voltage negative–positive slow wave without a spindle, delta sequence, another KC, or alpha/beta activity within ±2 s. Horizontal bar = 1 s (software 1 s grid). Amplitude follows the clinical display; scored KCs are ≥75 µV. A voltage calibration pulse was not stored in the export.

**Figure 2:**
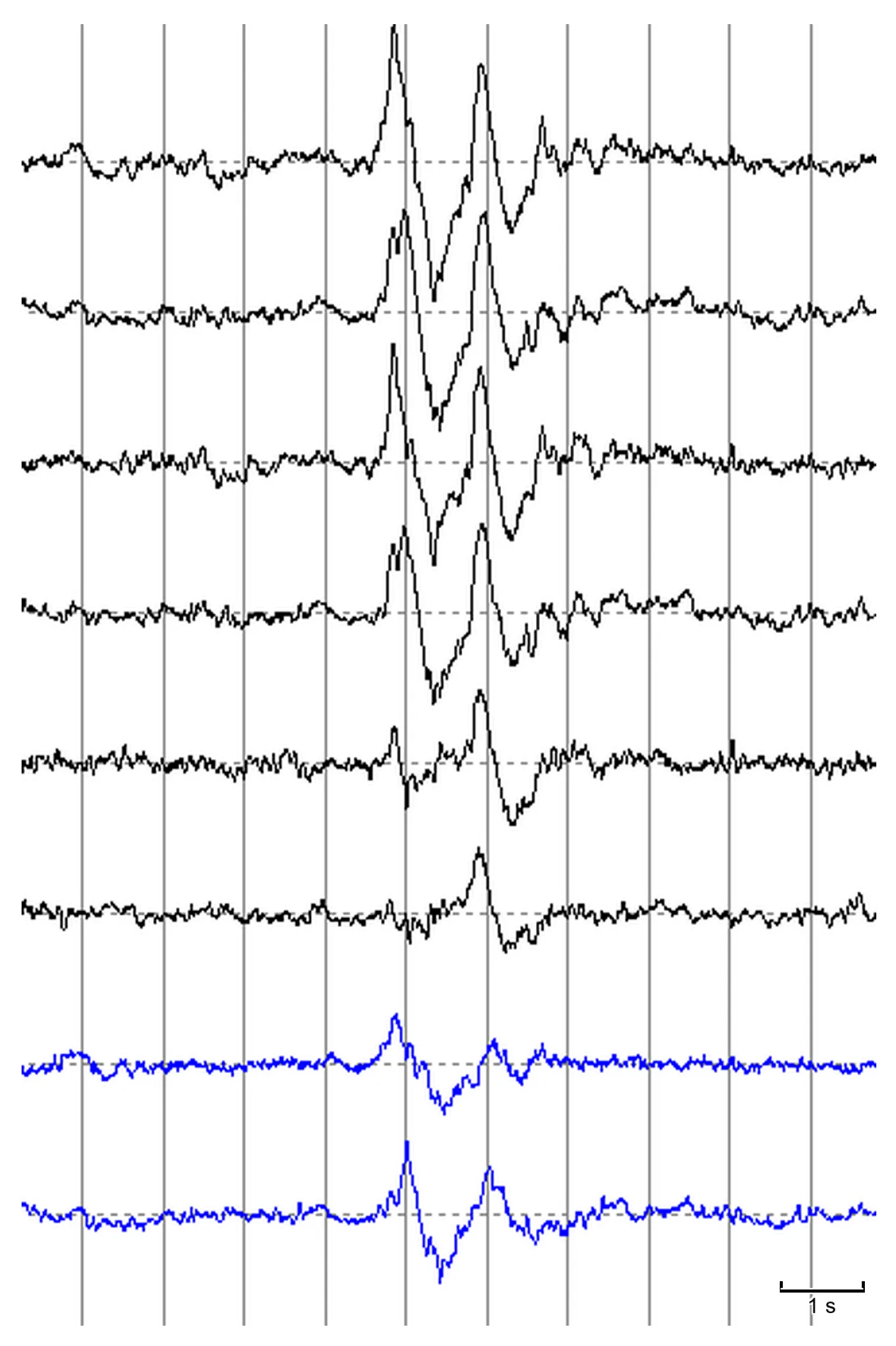
KC-Burst. Same montage as Figure 1 (EEG black; EOG blue). At least two KCs occur within 3 s, without alpha. Horizontal bar = 1 s (software 1 s grid). Amplitude follows the clinical display; scored KCs are ≥75 µV.

**Figure 3:**
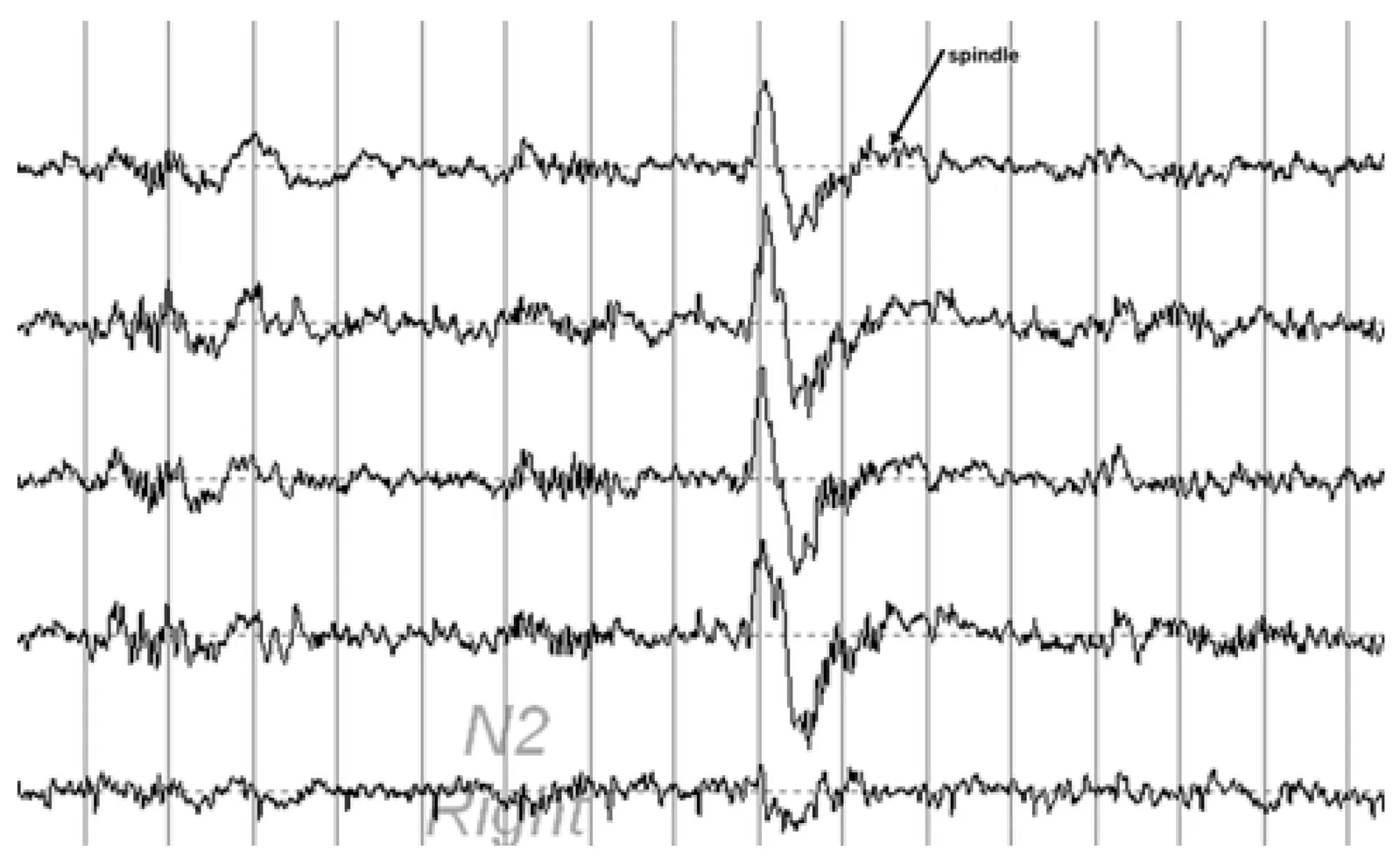
KC-Sigma. Same montage as Figure 1 (EEG black; EOG blue). The filled arrow marks the classifying sleep spindle (11–16 Hz) that overlaps or immediately follows the KC. Horizontal bar = 1 s (software 1 s grid). Amplitude follows the clinical display; scored KCs are ≥75 µV.

**Figure 4:**
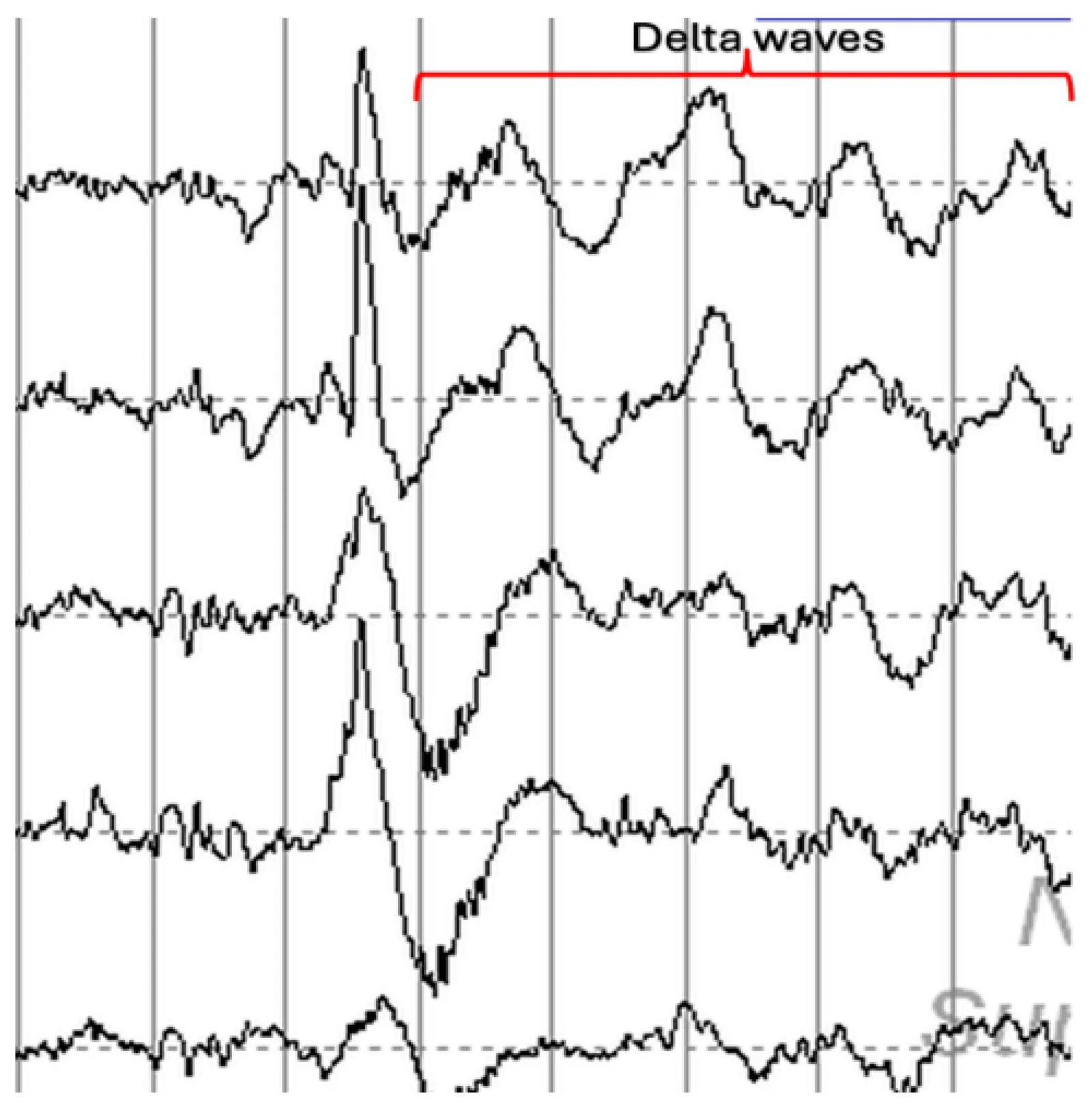
KC-Delta. Same montage as Figure 1 (EEG black; EOG blue). The brace labeled “Delta waves” marks the qualifying delta sequence (>2 s, at least two 0.5–4 Hz waves). A brief burst of fast activity near the KC that does not meet the spindle rule does not change the subtype. If a spindle and a qualifying delta sequence coexist, the priority rule assigns KC-Sigma rather than KC-Delta. Horizontal bar = 1 s (software 1 s grid). Displayed at the same time base as Figure 6. Amplitude follows the clinical display; scored KCs are ≥75 µV.

**Figure 5:**
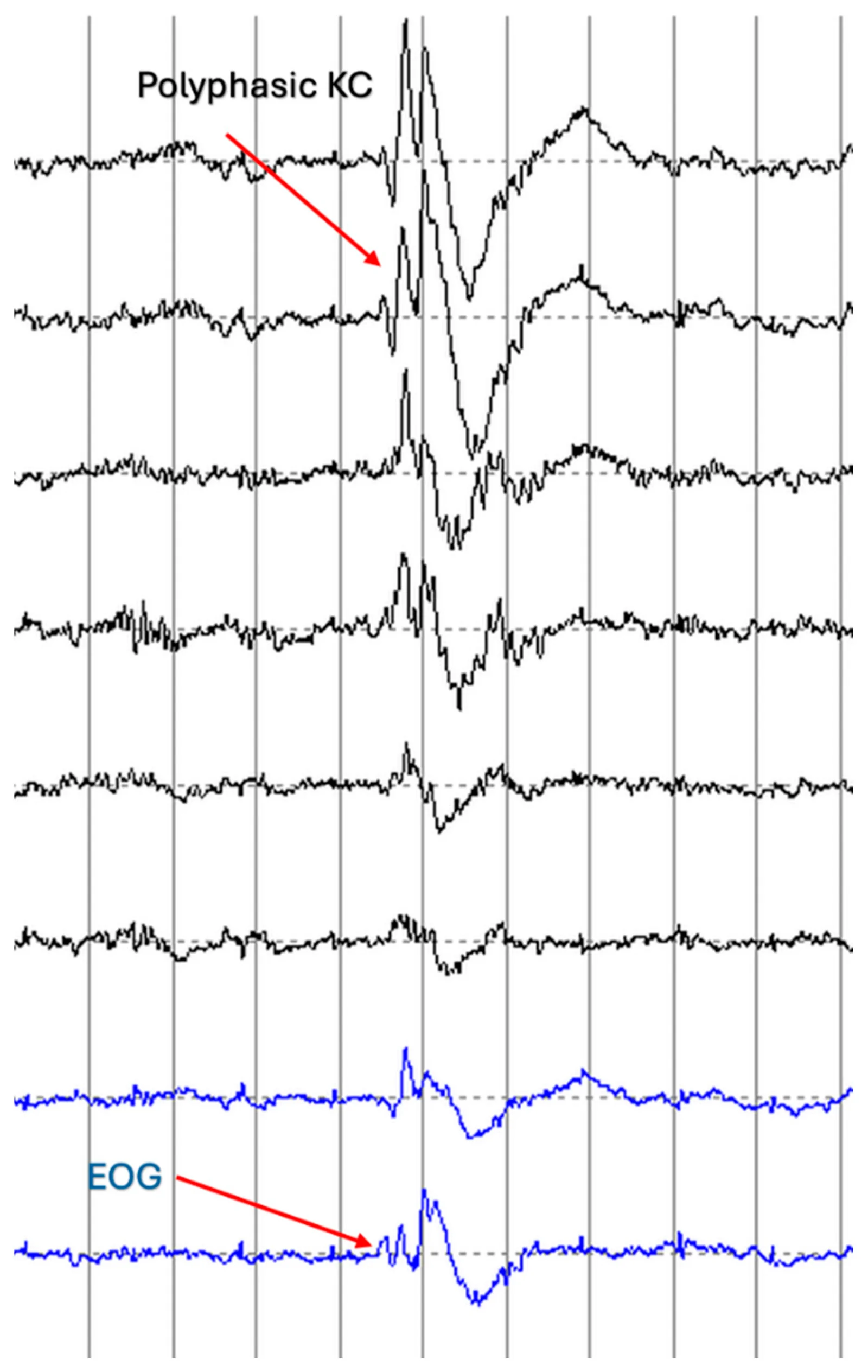
Polyphasic-KC. Same montage as Figure 1 (EEG black; EOG blue). The arrow labeled “Polyphasic KC” marks the extra high-voltage deflection on the initial negative phase, producing a triphasic/polyphasic contour. These deflections are interpreted as a KC variant, not as evidence that a vertex sharp wave and a KC occurred together. Horizontal bar = 1 s (software 1 s grid). Amplitude follows the clinical display; scored KCs are ≥75 µV.

**Figure 6:**
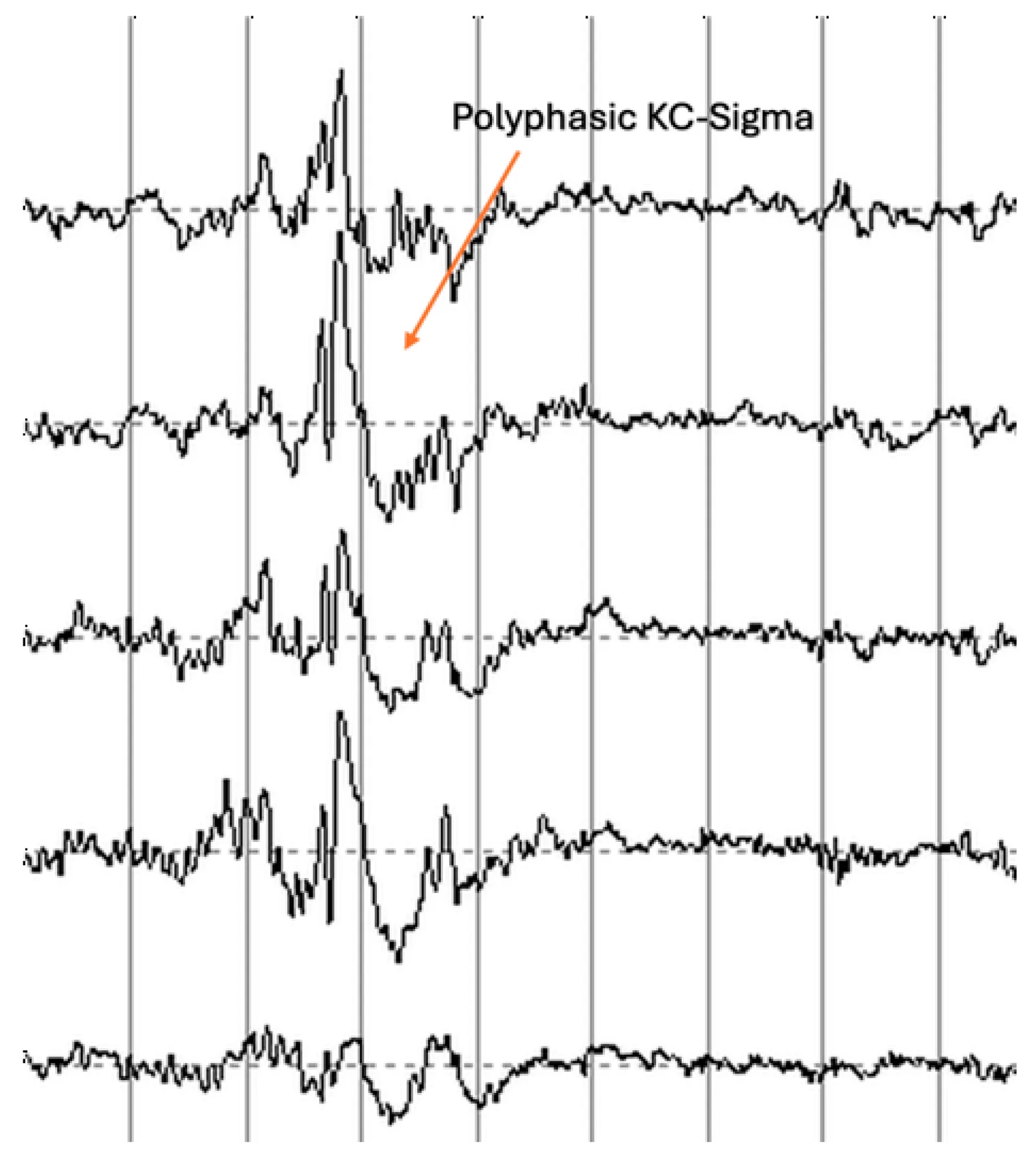
Polyphasic-KC-Sigma. Same time base as Figure 4. This excerpt shows the EEG derivations used for morphological classification; EOG traces are not displayed. The arrow labeled “Polyphasic KC-Sigma” marks the polyphasic contour together with the classifying spindle. A slower wave after the KC is visible but does not meet the KC-Delta rule (≥2 delta waves over >2 s in ≥2 derivations), so the event was not counted as KC-Delta. The label follows the priority hierarchy. Horizontal bar = 1 s (software 1 s grid). Amplitude follows the clinical display; scored KCs are ≥75 µV.

**Figure 7:**
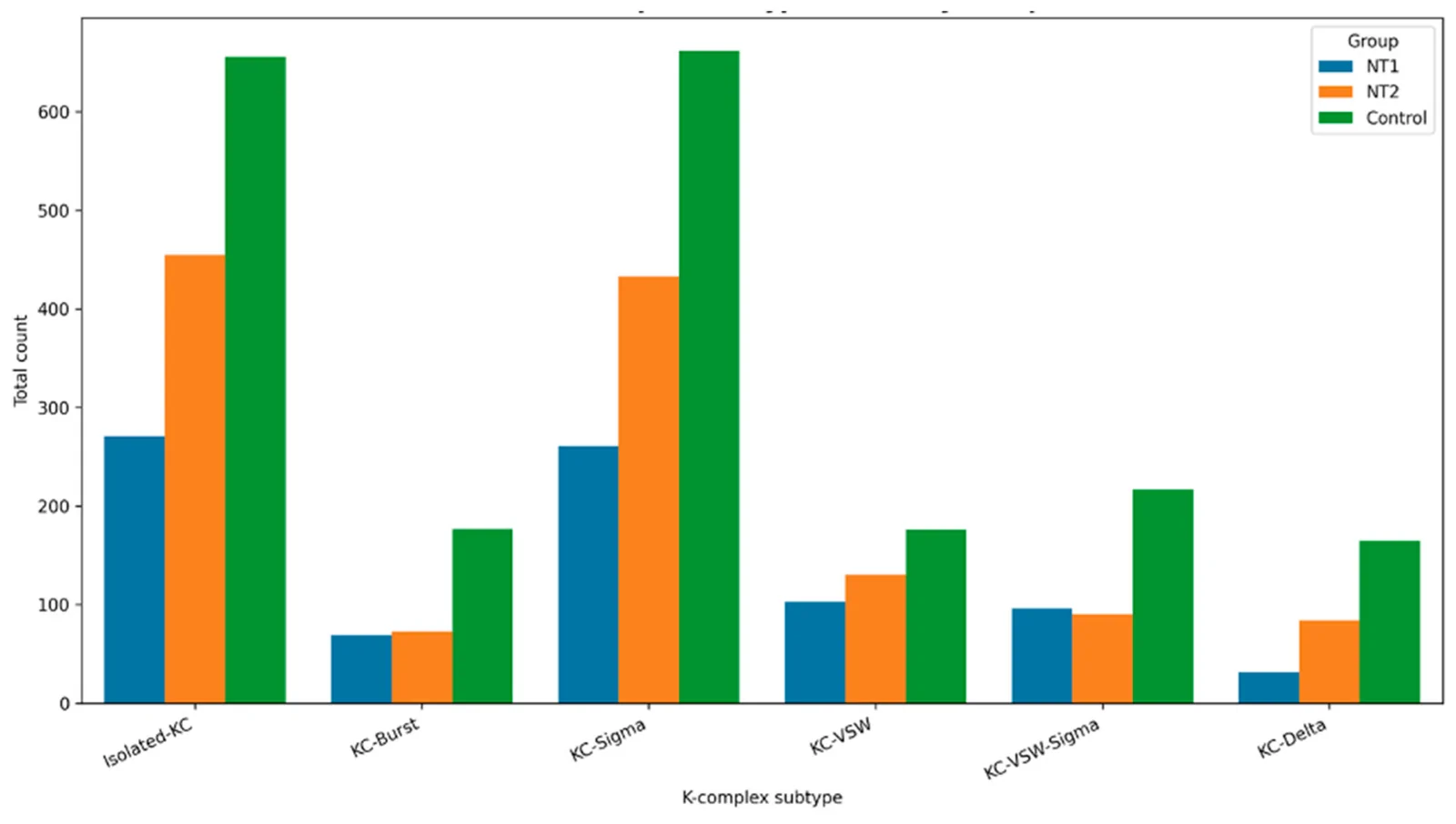
Total counts of K-complex subtypes in NT1 (*n* = 18), NT2 (*n* = 13), and controls (*n* = 15). Axis labels use the revised names Polyphasic-KC and Polyphasic-KC-Sigma (former KC-VSW and KC-VSW-Sigma). Corresponding inferential statistics are in Table 1.

Figure 1, Figure 2, Figure 3, Figure 4, Figure 5 and Figure 6 illustrate the six visually defined KC subtypes. Kruskal–Wallis tests on total subtype counts showed group differences for Isolated-KC, KC-Burst, KC-Sigma, KC-Delta, and Polyphasic-KC-Sigma, but not for Polyphasic-KC (Figure 7; Table 1). For Isolated-KC, KC-Burst, KC-Sigma, and KC-Delta, control counts were highest and NT1 counts were lowest, with NT2 intermediate. For Polyphasic-KC-Sigma, control counts were highest, whereas NT1 and NT2 totals were similar (NT1 96, NT2 90). Polyphasic-KC did not differ among groups (*p* = 0.098). The lowest overall totals were in KC-Delta.

### 4.3. Cycle-Period Comparisons

**Figure 8:**
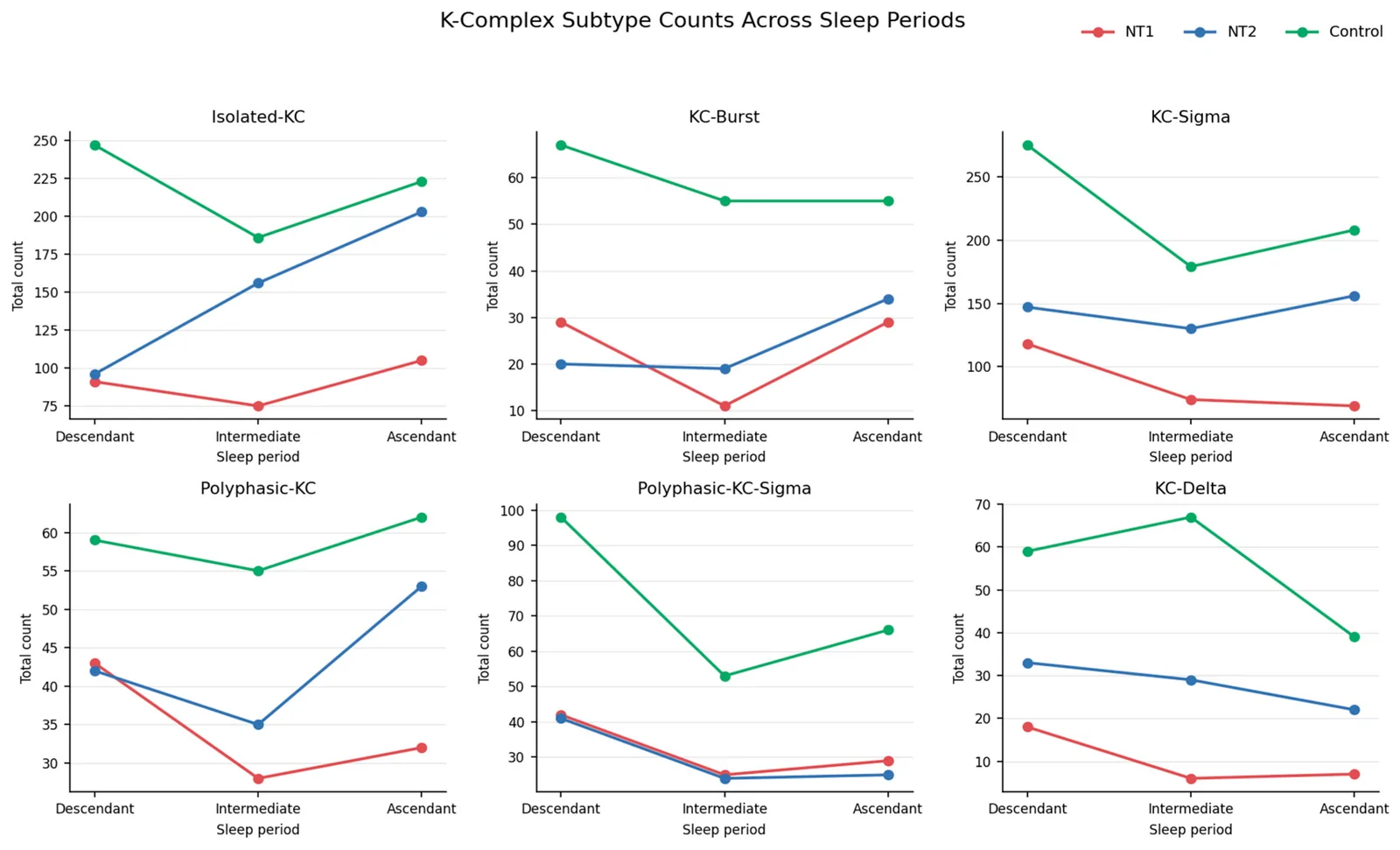
K-complex subtype counts across Descendant, Intermediate, and Ascendant periods in NT1, NT2, and controls. Panel titles use the revised names Polyphasic-KC and Polyphasic-KC-Sigma (former KC-VSW and KC-VSW-Sigma). Pairwise *p*-values are listed in Table 2.

Pairwise contrasts by cycle period are illustrated in Figure 8 and listed in Table 2. Isolated-KC and KC-Sigma were lower in NT1 than in controls in the Descendant, Intermediate, and Ascendant periods. KC-Delta was lower in NT1 than in controls in all three periods, and lower in NT1 than in NT2 in the Descendant (*p* = 0.030) and Intermediate (*p* = 0.015) periods. KC-Sigma was also lower in NT1 than in NT2 in the Descendant period (*p* = 0.041). Isolated-KC was lower in NT1 than in NT2 in the Intermediate period (*p* = 0.039). These four cells are the only NT1–NT2 contrasts in Table 2 with *p* < 0.05. NT2 differed from controls only for Descendant Isolated-KC (*p* = 0.047). Smaller period *n* than the global KC *n* reflects nights without a scorable D, I, or A segment, not exclusion of additional participants.

### 4.4. Overnight Sleep Architecture (NT1 Versus NT2)

**Figure 9:**
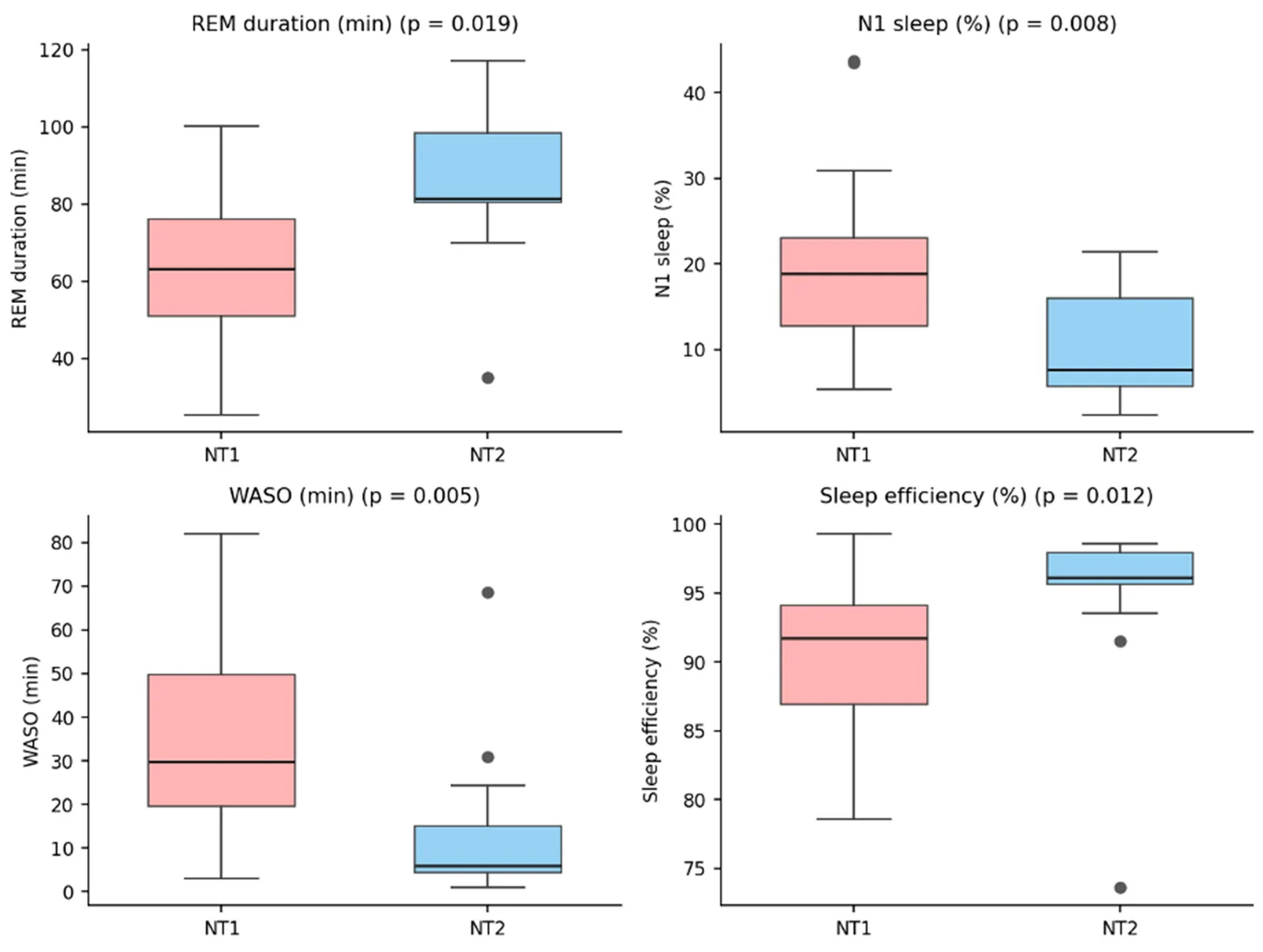
PSG variables that differed between NT1 and NT2 (Table 3): shorter REM duration (*p* = 0.019), higher N1 percentage (*p* = 0.008), longer WASO (*p* = 0.005), and lower sleep efficiency (*p* = 0.012) in NT1. Complete overnight PSG summaries were available for 16 NT1 and 13 NT2 participants. Sleep efficiency uses the corrected NT1 value of 99.3% in place of the archived 0.1% entry.

Complete overnight PSG summaries were available for 16 NT1 and 13 NT2 participants (Figure 9; Table 3). Compared with NT2, NT1 had shorter REM duration (63.3 ± 21.3 versus 85.1 ± 20.6 min; *p* = 0.019), a higher N1 percentage (20.2 ± 11.2% versus 10.3 ± 6.0%; *p* = 0.008), longer WASO (34.7 ± 21.1 versus 14.2 ± 18.6 min; *p* = 0.005), and lower sleep efficiency (90.7 ± 5.4% versus 94.6 ± 6.6%; *p* = 0.012). These macrostructural differences are reported because they may influence the amount of scorable N2 and therefore KC counts; they were not modeled as mediators. One archived NT1 sleep-efficiency entry of 0.1% was replaced by the efficiency implied by that night’s stage durations and WASO (99.3%). Table 4 summarizes the clinical characteristics of the K-complex-scored sample.

## 5. Discussion

Using a six-subtype visual KC scheme (Figure 1, Figure 2, Figure 3, Figure 4, Figure 5, Figure 6 and Figure 7; Table 1), NT1 showed lower Isolated-KC and KC-Sigma counts than controls across cycle periods (Figure 8; Table 2). NT1 participants with complete overnight PSG summaries also had more fragmented sleep than NT2 (higher WASO and N1 percentage, lower sleep efficiency; Figure 9; Table 3; *n* = 16 vs. *n* = 13). The NT1–NT2 morphological contrasts that reached *p* < 0.05 were KC-Delta in the Intermediate (*p* = 0.015) and Descendant (*p* = 0.030) periods, Isolated-KC in the Intermediate period (*p* = 0.039), and KC-Sigma in the Descendant period (*p* = 0.041). KC-Delta is the only subtype that differed between NT1 and NT2 in two cycle periods; Isolated-KC and KC-Sigma each differed in one period. These observations are compatible with more disrupted N2 microstructure in NT1, but they do not prove a specific hypocretin-dependent mechanism and should not be read as a diagnostic biomarker.

The sleep-protective and arousal-related readings of the KC remain a useful clinical frame [6,7,29]. In this dataset, lower Isolated-KC and KC-Sigma counts in NT1 sat alongside worse sleep continuity. That association is descriptive. We did not test whether KC loss causes awakenings, or whether awakenings simply leave less stable N2 in which KCs can be scored. The lower KC-Sigma count in NT1 than in NT2 during the Descendant period is consistent with previous reports that spindle-related activity can differ between narcolepsy subtypes [27], but we did not perform spectral spindle analysis.

The etiology of NT2 is still uncertain. Preservation of KC-Delta in NT2 relative to NT1 is a compact group difference in this sample, not a validated marker of residual hypocretin function [23,24,25]. CSF hypocretin-1 was not measured here, so any link to hypocretin status is inferential only.

Earlier classifications (Johnson and Karpan; Paiva and Rosa; Ujszászi and Halász; CAP) are also morphological, and several of them are more finely divided than ours [10,11,12,13]. We do not argue that the Gülhane scheme is inherently better. Its practical difference is narrower: mutually exclusive visual labels plus D/I/A period assignment on routine overnight PSG. Polyphasic-KC (formerly called KC-Vertex) did not differ among groups (*p* = 0.098). Its nature is unknown, and that part of the classification is therefore down-weighted in this revision. KC-Delta, not Polyphasic-KC, had the lowest subtype totals.

Limitations follow directly from the archive. The study is retrospective and single-center. The narcolepsy groups were entirely male, so sex effects on KC microstructure could not be tested, and BMI was unavailable. Observed ages were 15–41 years in NT1, 22–46 years in NT2, and 17–39 years in controls; because KC morphology changes with age, residual age confounding is possible, and two participants were younger than 18. Controls were simple snorers rather than asymptomatic healthy sleepers, so snoring-related KCs may have inflated control counts. CSF hypocretin-1, detailed lifetime treatment exposure, and formal inter-rater kappa were not available. Subtype assignment was visual, not spectral. Three PSG platforms were used without cross-device recalibration. Prospective studies with sex-balanced adult samples, BMI and treatment recording, hypocretin assay when indicated, and reported scoring reliability are required before clinical use can be considered. Pairwise subtype-by-period tests were not corrected for multiple comparisons, so isolated *p* < 0.05 cells should be read as unadjusted. One NT1 sleep-efficiency value archived as 0.1% was replaced by 99.3% computed from the same night’s stage durations and WASO. Per-event KC phase durations (negative-phase length, positive-phase length, and their sum) were not exported from the scoring archive; the ≥0.5 s rule was applied visually for event identification, and duration was not an analyzed endpoint.

## 6. Conclusions

In this retrospective PSG series, Isolated-KC and KC-Sigma counts were lower in NT1 than in controls across NREM-cycle periods. NT1 recordings with complete PSG summaries also showed more fragmented overnight sleep than NT2. Between NT1 and NT2, KC-Delta differed in two cycle periods, and Isolated-KC and KC-Sigma each differed in one period. The Gülhane labels are an operational visual aid, not a replacement for ICSD-3 diagnosis, MSLT, or hypocretin testing. Independent, prospective replication is needed before any clinical application is proposed.

## Figures and Tables

**Table 1:** Global comparison of K-complex subtypes (Kruskal–Wallis; NT1 *n* = 18, NT2 *n* = 13, control *n* = 15). Significance: *** *p* < 0.001, ** *p* < 0.01, ns = not significant. Significant group differences were present for all subtypes except Polyphasic-KC.

Subtype	Kruskal–Wallis Statistic	*p*-Value	Significance
KC-Sigma	26.00	0.00000	***
Isolated-KC	23.75	0.00001	***
KC-Delta	22.03	0.00002	***
KC-Burst	19.25	0.00007	***
Polyphasic-KC-Sigma	11.32	0.00349	**
Polyphasic-KC	4.64	0.09849	ns

**Table 2:** Post hoc pairwise comparisons by sleep period (Descendant, Ascendant, Intermediate). Significance: ** *p* < 0.01, * *p* < 0.05. C = control. Isolated-KC and KC-Sigma distinguish NT1 from controls in all three periods. NT1–NT2 contrasts with *p* < 0.05 are Descendant KC-Delta (*p* = 0.030), Descendant KC-Sigma (*p* = 0.041), Intermediate Isolated-KC (*p* = 0.039), and Intermediate KC-Delta (*p* = 0.015). Pairwise *n* is the available-period *n*, not the global scoring *n*. *p*-values are uncorrected. Period *n* is smaller than the global KC *n* when a night lacks that segment (see Methods).

Phase	Subtype	Comparison	Comparison2 (Count)	*p*-Value
Descendant (D)	Isolated-KC	NT1 vs. C	C > NT1	0.002 **
	Isolated-KC	NT2 vs. C	C > NT2	0.047 *
	KC-Delta	NT1 vs. C	C > NT1	0.023 *
	KC-Delta	NT1 vs. NT2	NT2 > NT1	0.030 *
	KC-Sigma	NT1 vs. C	C > NT1	0.004 **
	KC-Sigma	NT1 vs. NT2	NT2 > NT1	0.041 *
	Polyphasic-KC-Sigma	NT1 vs. C	C > NT1	0.008 **
Ascendant (A)	Isolated-KC	NT1 vs. C	C > NT1	0.010 *
	KC-Burst	NT1 vs. C	C > NT1	0.011 *
	KC-Delta	NT1 vs. C	C > NT1	0.002 **
	KC-Sigma	NT1 vs. C	C > NT1	0.001 **
Intermediate (I)	Isolated-KC	NT1 vs. C	C > NT1	0.008 **
	Isolated-KC	NT1 vs. NT2	NT2 > NT1	0.039 *
	KC-Burst	NT1 vs. C	C > NT1	0.004 **
	KC-Delta	NT1 vs. C	C > NT1	0.010 *
	KC-Delta	NT1 vs. NT2	NT2 > NT1	0.015 *
	KC-Sigma	NT1 vs. C	C > NT1	0.007 **

Abbreviations: KC: K Complex, NT1: Narcolepsy Type 1, NT2: Narcolepsy Type 2; C: Control.

**Table 3:** NT1 versus NT2 polysomnography parameters (Mann–Whitney U; NT1 *n* = 16, NT2 *n* = 13). Significance: ** *p* < 0.01, * *p* < 0.05. One NT1 sleep-efficiency value archived as 0.1% was replaced by 99.3%, computed from that record’s stage durations and WASO.

Parameter	NT1 Mean ± SD	NT2 Mean ± SD	*p*-Value	Significance
REM duration (min)	63.3 ± 21.3	85.1 ± 20.6	0.019	*
N1 sleep (%)	20.2 ± 11.2	10.3 ± 6.0	0.008	**
WASO (min)	34.7 ± 21.1	14.2 ± 18.6	0.005	**
Sleep efficiency (%)	90.7 ± 5.4	94.6 ± 6.6	0.012	*

Abbreviations: NT1: Narcolepsy Type 1, NT2: Narcolepsy Type 2, WASO: Wake After Sleep Onset.

**Table 4:** Clinical characteristics of the K-complex-scored sample (NT1 *n* = 18, NT2 *n* = 13, control *n* = 15). Values are mean ± SD or *n*. NA = not available in the archive. AHI was <5 in all included recordings. Controls were simple snorers. CSF hypocretin-1 was not measured. Overnight PSG-summary *n* (NT1 *n* = 16, NT2 *n* = 13) is stated in Table 3.

Variable	NT1	NT2	Control
*n*	18	13	15
Age, years	25.4 ± 7.0 (15–41) †	31.0 ± 7.6 (22–46)	25.6 ± 7.1 (17–39) †
Male/female, *n*	18/0	13/0	12/3
Participants < 18 years, *n*	1	0	1
Disease duration, years	8.6 ± 4.8 (*n* = 14)	9.8 ± 6.6 (*n* = 13)	NA
ESS	17.5 ± 3.8 (*n* = 11)	17.5 ± 2.8 (*n* = 12)	NA
AHI	<5	<5	<5 (simple snoring)
BMI	NA	NA	NA
CSF hypocretin-1	Not measured	Not measured	Not measured
Current REM-suppressants/antidepressants/antipsychotics	Excluded	Excluded	Excluded
Inclusion period	December 2016–January 2026	December 2016–January 2026	December 2016–January 2026

† Age available for 17 of 18 NT1 and 11 of 15 controls. ESS = Epworth Sleepiness Scale. NA = not available. A complete lifetime treatment inventory was not uniformly recorded.

## Data Availability

Summary counts and group statistics supporting the results are presented in the tables. Individual-level polysomnography recordings are not publicly deposited because they contain identifiable clinical data; reasonable requests may be directed to the corresponding author, subject to ethics-committee approval.
